# Genetic Analysis of Vitamin C Content in Rapeseed Seedlings by the Major Gene Plus Polygene Mixed Effect Model

**DOI:** 10.3390/cimb46090568

**Published:** 2024-08-29

**Authors:** Chao Wang, Tao Wang, Xinfa Wang, Hanzhong Wang, Xiaoling Dun

**Affiliations:** 1 Key Laboratory of Biology and Genetic Improvement of Oil Crops, Oil Crops Research Institute of the Chinese Academy of Agricultural Sciences, Ministry of Agriculture, Wuhan 430062, China; wangchao05360536@163.com (C.W.); wangxinfa@caas.cn (X.W.); wanghanzhong@caas.cn (H.W.); 2 Guizhou Rapeseed Institute, Guizhou Academy of Agricultural Science, Guiyang 550007, China; wangtao10062023@163.com

**Keywords:** rapeseed seedlings, vitamin C, major gene, polygene, inheritance

## Abstract

Rapeseed (*Brassica napus* L.) seedlings are rich in vitamin C (Vc), which is beneficial for humans. Understanding the genetic variance in Vc content has practical significance for the breeding of “oil–vegetable dual-purpose” rapeseed. In this study, the joint segregation analysis of a mixed genetic model of the major gene plus polygene was conducted on the Vc content in rapeseed seedlings. Six generations, including two parents, P_1_ (high Vc content) and P_2_ (low Vc content), F_1_, and the populations of F_2_, BC_1_P1, and BC_1_P_2_ from two crosses were investigated. Genetic analysis revealed that the genetic model MX2-A-AD was the most fitting genetic model, which indicates that Vc content is controlled by two additive major genes plus additive and dominance polygenes. In addition, the whole heritability in F_2_ and BC_1_P1 was higher than that in BC_1_P2. The largest coefficient of variation for Vc content appeared in the F_2_ generation. Therefore, for Vc content, the method of single cross recross or single backcross are suggested to transfer major genes, and the selection in F_2_ would be more efficient than that in other generations. Our findings provide a theoretical basis for the quantitative trait locus (QTL) mapping and breeding of Vc content in rapeseed seedlings.

## 1. Introduction

Rapeseed, also known as *Brassica napus* L. (AACC, 2n = 38), is a crucial allotetraploid plant, derived from the hybridization of *Brassica rapa* (AA) and *Brassica oleracea* (CC,); it serves as the third-largest source of vegetable oil globally [1,2]. Although the rapeseed industry in China has made great progress in recent years, the economic benefits of rapeseed are not high. Except for oil, rapeseed also has multiple functions, such as a vegetable, flower, honey, forage, and fertilizer [3]. Moreover, the planting efficiency of rapeseed can be greatly improved through “oil–vegetable dual-purpose” (OVDP), which is one of the important examples of multifunctional utilization of rapeseed in China [4,5]. In addition, our previous research found that rapeseed seedlings and rapeseed flower stalks are rich in vitamin C (Vc), selenium (Se), zinc (Zn), and other nutrients that have high edible value.

Vc, commonly known as L-ascorbic acid (AsA), is an essential metabolite in both plants and animals [6]. Numerous studies have demonstrated the role of Vc in preventing various illnesses related to oxidative stress, including cancer [7,8], cardiovascular diseases [9,10], aging [11,12], and other inflammatory diseases [13,14]. In the Institute of Medicine regulations, (Bethesda, MD, USA), it is recommended that adults consume 90 mg of Vc daily for men and 75 mg for women [15]. As an essential micro-nutrient that humans cannot synthesize, it must be acquired through dietary uptake, primarily from fresh fruits and vegetables [16,17]. In plants, Vc possesses significant reactive oxygen species (ROS) scavenging capabilities and impacts several key plant functions, such as photosynthesis, respiration, cell expansion and division, growth regulation, plant development, hormone signaling, senescence, and abiotic stress responses (such as drought, high temperature, cold damage, and salinity) [18,19,20,21,22,23]. Understanding the biosynthetic pathway of Vc and identifying the factors that regulate its levels in edible plant organs is fundamental for the enhancement of Vc levels in fruits and vegetables.

In recent years, the investigation of Vc levels in crops has gained more attention. In studies in various plants, including *Arabidopsis* [24,25,26], tomato [27,28,29], kiwifruit [30,31,32], apple [33], and strawberry [34], the genetic basis of Vc has been determined, which contributed significantly to the molecular breeding of crop nutritional quality. However, the nutritional analysis of rapeseed as a new vegetable, particularly regarding the genetic mechanism of Vc content, has been lacking. Therefore, the foundation for the effective and high-quality production of edible rapeseed will be laid by carrying out research to fully comprehend the genetic metabolism mechanism of Vc in rapeseed seedlings.

Various methods, such as bulked segregant analysis (BSA), mutant analysis, genome-wide association studies (GWAS), and quantitative trait loci mapping (QTL), have been commonly utilized to reveal the genetic makeup of variations crucial for breeding purposes [35]. A method of analysis utilizing a model that combines major gene and polygenes effects has been formulated for plants [36]. This mixed model has been extensively applied to different crop species, such as rice [37], cotton [38], soybean [39], rapeseed [40], non-heading Chinese cabbage [41], tomato [42], eggplant [43], jujuba [44], bearded iris [45], crape myrtle [46], and wolfberry [47], for assessing superior agronomic traits through genetic analysis. The inferences obtained through the major genes plus polygenes model have shown consistency with those yielded by QTL analysis, signifying its utility as an effective and cost-effective approach for investigating intricate quantitative traits [48].

In the present study, we used high-Vc content varieties and low-Vc content varieties as parental lines in two breeding crosses. Through six generations (P_1_, P_2_, F_1_, F_2_, BC_1_P_1_, and BC_1_P_2_), a diverse genetic pool was obtained. The genetic model combining major gene effects with polygenic inheritance was employed to elucidate the genetic mechanisms governing the Vc content of rapeseed seedlings. Additionally, we assessed both major genetic contributions and overall genetic forces influencing Vc levels. These findings offer valuable insights for future QTL analysis, marker-assisted selection breeding programs, and the development of cultivars with enhanced Vc content in rapeseed.

## 2. Materials and Methods

### 2.1. Plant Material

The parent materials, *B. rapus* 8S079 and 8S243, selected for this study are high in Vc content, while *B. rapus* 8S007 and 8S084 are not. In October 2022, the parental materials were sown at the Yangluo Experimental Base of the Oil Crop Research Institute, Chinese Academy of Agricultural Sciences, Wuhan City, Hubei Province. In March 2023, two hybrid crosses were created through artificial emasculation and pollination: 8S079 (P_1_) × 8S084 (P_2_) (Cross A) and 8S243 (P_1_) × 8S007 (P_2_) (Cross B), also including their reciprocal crosses. In May 2023, the parental materials of the two hybrid crosses and their F_1_ generation were planted at the Ping’an Northern Propagation Experimental Base of the Chinese Academy of Agricultural Sciences in Qinghai Province. During the flowering period, F_1_ × P_1_ (BC_1_P_1_) and F_1_ × P_2_ (BC_1_P_2_) crosses were prepared. The F_1_ plant was used to self-cross to produce the F_2_ generation. Ultimately, seeds from six generations (P_1_, P_2_, F_1_, BC_1_P_1_, BC_1_P_2_, and F_2_) were obtained for the two hybrid crosses.

### 2.2. Growth Conditions

The six generations of the two hybrid crosses were cultivated in a greenhouse. Rapeseed seeds were initially placed on medical gauze within a germination device for 2 days in the dark at 24 °C, followed by growth under light conditions (180 µmol·m^−2^·s^−1^, 16/8 h) for 4 days in a greenhouse with 60–80% relative humidity and an air temperature of 24 ± 2 °C. One quarter of modified Hoagland’s nutrient solution was added to the germination device to maintain moisture and provide nutrients for seed germination. After six days, uniform seedlings were moved to a growth device with one quarter of Hoagland’s solution, which was later replaced with one half of Hoagland’s solution. Following 16 days of growth, samples of the above-ground tissue of rapeseed seedlings were collected to determine the Vc content.

### 2.3. Extraction and Determination of Vc Content

Samples of rapeseed seedlings were collected and promptly frozen in liquid nitrogen, and then ground into a fine powder. Approximately 1.0 g of ground sample was dissolved in a 25 mL solution of 0.1% hydrochloric acid solution and treated by a high shear dispersing emulsifier for 60 s. Next, the liquid was filtered through a 0.22 μm filter and 10.0 μL of the supernatant was injected into the HPLC-PDA system. The HPLC-PDA was performed using a Waters e2695 system (Milford, MA, USA) including a Waters e2695 Separations Module and a Waters 2998 UV-Vis Photo-diode Array Detector (PAD) with an autosampler. The HPLC-PDA system conditions were as follows: C18 column (250 mm × 4.6 mm × 5 μm, CNW) (Shanghai, China) with the temperature of 30 °C and detected at 245 nm wavelengths; methanol/20 mM ammonium acetate (3:97, *v*/*v*) as an affluent phase with the flow rate of 1.0 mL/min; retention time, 2.67 min (Figure S1). According to the standard curve of Vc content produced, it was used as a reference standard to quantify the Vc content in the rapeseed seedlings (Figure S2).

### 2.4. Statistical and Genetic Analysis

Statistical analysis was performed using SPSS 26.0 (IBM SPSS Statistics 26.0) statistical software. Frequency histograms were plotted using Origin 2024 (2024b, Origin Laboratory) software. In the figures, different letters above the bars indicate significant differences (*p* < 0.05) as determined by a one-way ANOVA test, the data represented the mean values, and the error bars represent the standard error of the mean. The R software package SEA v2.0 (https://cran.r-project.org/web/packages/SEA/index.html, accessed on 10 April 2024) was used to conduct a quantitative trait major gene plus polygene analysis on the Vc content of rapeseed seedlings across six generations [49]. The software for segregation analysis was provided by the team of the Crop Information Center, College of Plant Science and Technology, Huazhong Agricultural University, Wuhan, China. The analysis included 24 types of genetic models, with maximum likelihood values and Akaike’s information criterion (AIC) calculated. Two alternative models were chosen based on minimum AIC value and subjected to suitability tests, including uniformity tests (*U*_1_^2^, *U*_2_^2^, *U*_3_^2^), Smirnov’s statistics (n*W*^2^), and Kolmogorov’s statistics (*D*n). If no significant difference was found, the model with the smaller parameter value will be selected. Using the least-squares method, we estimated the genetic parameters of the optimal genetic model in the first and second orders. Afterward, based on the estimates of component distributions in the optimal genetic model, the genetic parameters pertaining to the gene effects, genetic variances, and heritability of the major genes were computed.

## 3. Results

### 3.1. Statistical Analysis of Vc Content in Six Generations from Two Crosses

**Figure 1:**
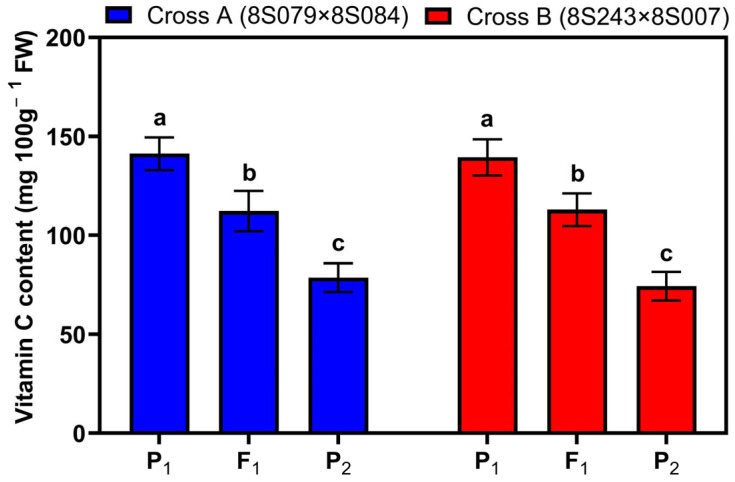
Comparison of Vc content among the parents (P_1_ and P_2_) and F_1_ generation of crosses A and B. Different letters above the bars indicate significant differences (*p* < 0.05) as determined by a one-way ANOVA test. FW, fresh weight.

As shown in Figure 1, there were significant differences in the Vc content among the two parents, P_1_ (high Vc content) and P_2_ (low Vc content), and the hybrid offspring (F_1_) generations of crosses A and B (*p* < 0.05), which enabled the segregation of vitamin C content in their offspring. The Vc content of F_1_ was intermediate between the two parents, with a slight bias towards the high-value parent. There was no significant difference in the Vc content of F_1_ between reciprocal crosses.

The results in Table 1 show that in crosses A and B, the BC_1_P_1_ generation exhibited the highest Vc content (125.52 and 122.81 mg/100 g, respectively) compared to the other offspring generations. The coefficient of variation (CV) varied from 9.11% to 20.07% and from 7.31% to 25.05% in the offspring generations of crosses A and B, respectively, indicating a wide range of phenotypic performances among the generations. Notably, the F_2_ generation of the two crosses showed the largest CV (20.07 and 25.05%, respectively) for Vc content. This segregation pattern in Vc content among the offspring made them suitable for further genetic analysis.

### 3.2. Distribution of Vc Content in Segregated Populations of Two Crosses

**Figure 2:**
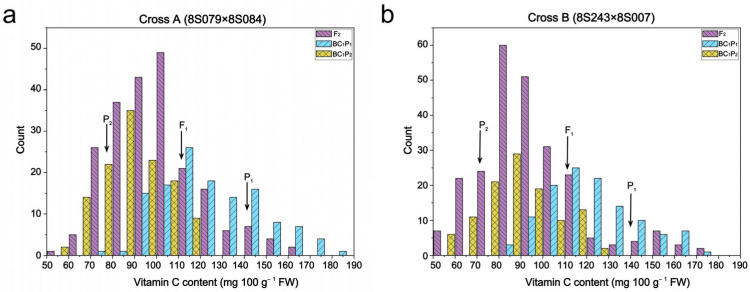
Frequency distributions of style Vc content in four generations of two crosses. (**a**) Cross A (8S079 × 8S084); (**b**) cross B (8S243 × 8S007). FW, fresh weight.

In cross A, the skewness and kurtosis values of the Vc content in the generations of BC_1_P_1_, BC_1_P_2_, and F_2_ were all smaller than 1. A similar phenomenon was identified in cross B, except for the kurtosis values of F_2_ (Table 2). Generally, these data suggest that the distributions of the Vc content in the three hybrid offspring followed a normal pattern. Figure 2 shows the frequency distributions of the Vc content across the four offspring generations in the two crosses. The generation of BC_1_P_1_ showed a skewed content of Vc towards P_1_ in both crosses, while BC_1_P_2_ was skewed towards P_2_. These findings demonstrate that the genetic characteristics of Vc content in rapeseed seedlings follow a typical quantitative trait pattern, suggesting a genetic model of the major gene plus polygene interactions influencing Vc content, which could be valuable in selecting superior individuals in breeding programs.

### 3.3. Selection and Testing for the Best Genetic Model of Vc Content

The Vc content of rapeseed seedlings was analyzed for the six generations using a major gene plus polygene mixed genetic model. Then, 24 types of genetic models were evaluated by calculating values for the maximum likelihood method and Akaike’s information criterion (AIC) (Table 3). A lower AIC value indicates that the model offers a more precise representation of the data, implying that its predictive error is minimized. The model with the minimum AIC value was determined to be the best candidate model [41]. Among the 24 genetic models, two genetic models were chosen as potential candidate models based on their relatively low AIC values. In both crosses A and B, the genetic model of MX2-A-AD had the lowest estimated AIC value, followed by MX2-ADI-AD and MX2-ADI-ADI, respectively.

Furthermore, tests of goodness-of-fit were performed for the candidate models to determine the most suitable one. Then, five statistical parameters were evaluated, including equal distribution (*U*_2_^1^, *U*_2_^2^, and *U*_2_^3^), Smirnov (n*W*^2^), and Kolmogorov tests (*D*n). The results of the goodness-of-fit tests for the two crosses are presented in Table 4. The model that achieved significance with the smallest number of statistics (*p* < 0.05) was chosen as the most optimal model [42]. When no significant differences were observed among the candidate models, the preferred model was selected based on the lowest AIC value. The results suggest that the statistical comparison between the two models in crosses A and B did not show significant differences.

Based on the lowest AIC value, MX2-A-AD was chosen as the optimal genetic model for the Vc content in rapeseed seedlings. This suggests that the Vc content in rapeseed seedlings was affected by two major additive genes plus additive and dominant polygenic effects.

### 3.4. Estimation of Genetic Parameters for the Optimal Genetic Model of Vc Content

Table 5 lists the genetic models of the first-order parameters related to Vc content in the MX2-A-AD genetic model of crosses A and B. The study indicated that interactions between two pairs of major genes influenced the Vc content, with the additive effects of the first major gene being more significant than those of the second major gene. The additive effects of the two gene pairs were 22.43 and −6.34, and 30.72 and −9.61, respectively. The above results show that the inheritance of Vc content was primarily driven by the additive effect of the first pair of major genes, resulting in a positive synergistic effect. Furthermore, the additive and dominant effects of the polygenes were 19.65 and −0.52, and 15.95 and 3.20, respectively. The dominance of the additive effects of polygenes in the inheritance of Vc content was highlighted by the fact that the additive effect was greater than the dominant effect.

The genetic parameters in the second order of the optimal genetic model for Vc content in rapeseed seedlings were analyzed, as shown in Table 6. The heritabilities of the BC_1_P_1_, BC_1_P_2_, and F_2_ segregating generations resulting from crosses A and B are reported as follows: 86.60% and 82.83%, 66.97% and 76.69%, and 82.50% and 88.00%, respectively. Among these, the major genes contributed heritabilities of 42.05% and 74.15%, 54.92% and 51.52%, and 82.50% and 88.00%, respectively, while the polygenes accounted for 44.55% and 8.68%, 12.05% and 25.48%, and 0.00% and 0.00%, respectively. The major gene responsible for Vc content exhibited the highest heritabilities in the BC_1_P_1_ and F_2_ generations. Moreover, the environmental variance in the segregating generations of crosses A and B, including BC_1_P_1_, BC_1_P_2_, and F_2_, was estimated at 71.93 and 67.07, respectively. This estimation was based on P_1_, P_2_, and F_1_, with contributions of 13.40% and 17.17%, 33.03% and 23.31%, and 17.50% and 12.00%, respectively, to the phenotypic variance. These findings indicate that genetic factors play a significant role in controlling the Vc content of rapeseed seedlings, while environmental factors also have a substantial impact.

## 4. Discussion

Rapeseed (*B. napus* L.) evolved from the hybridization and doubling of two basic species, *B. rapa* and *B. oleracea* [50]. *Brassica* species have been associated with many beneficial health effects recently, such as potential anti-carcinogenic properties and the ability to protect against cardiovascular illnesses, aging, prenatal disorders, etc. [51,52,53,54,55]. The abundance of health-promoting phytochemicals such as carotenoids, phenolic compounds, glucosinolates, vitamins, and minerals is responsible for these advantages [56,57,58,59]. Vc is widely favored by the general public for its antioxidant properties. Cruciferous plants contain a variety of health-promoting compounds and are recognized as rich sources of Vc [60]. Studies have shown that Vc content is a crucial quality and agronomic trait in non-heading Chinese cabbage, which is influenced by multiple genes, making it a quantitative trait with a complex genetic mechanism [61]. Furthermore, Vc content could serve as an indicator for the quality breeding of OVDP rapeseed. However, until now, no research on Vc in rapeseed has been reported.

In this study, joint segregation analysis was utilized to analyze the inheritance and gene effects on Vc content in rapeseed seedlings across six generations from two crosses (8S079 × 8S084 and 8S243 × 8S007). The results show that the Vc content in rapeseed seedlings is governed by two major genes with additive effects. Similar to the inheritance patterns observed in vegetables and fruits, rapeseed seedlings exhibit complex genetic patterns of Vc content. The analysis of inheritance for plant quantitative traits is often conducted using a combination of major gene and polygene inheritance. In recent years, this approach has been applied to analyze the vitamin E content in soybeans [62], the chlorophyll content in maize [63], the vitamin P content in eggplant [43], the Vc content in non-heading Chinese cabbage [64] and pepper [65], and the fruit length in cucumbers [66]. Some studies have shown that the Vc content of certain plants was found to involve a pair of major genes. For example, in non-heading Chinese cabbage, the Vc content was controlled by a pair of additive major genes and additive dominant polygenes, as revealed by the joint analysis of six generations [64]. Similarly, the optimum model for the Vc content in cucumber was determined to be a combination of additive major genes and additive dominant polygenes [67]. However, the genetic model for Vc content in rapeseed seedlings in this study is inconsistent with other crops, which could be attributed to variations in the genetic background and biological environment of the research materials, resulting in potential biases in the estimation of model parameters and genetic parameters. Additionally, it is possible that the observed inconsistency is influenced by more intricate genetic regulations.

The heritabilities of major genes for Vc content in the BC_1_P_1_, BC_1_P_2_, and F_2_ generations of two crosses were estimated to range from 42.05% to 82.50% and 51.21% to 88.00%, respectively. The environmental variance in phenotypic variance for the two crosses was estimated to range from 13.40% to 33.03% and 12.00% to 23.31%, respectively, indicating a significant role of environmental factors in the genetic Vc content of rapeseed seedlings. The results reveal that the overall heritability of Vc content in the F_2_ and BC_1_P_1_ generations was consistently higher than that in the BC_1_P_2_ generation for each cross. Moreover, the F_2_ generation of both crosses exhibited the largest coefficient of variation for Vc content, suggesting that selection in F_2_ could breed rapeseed germplasm with a higher Vc content. Based on the joint segregation analysis of phenotypic data, the study identified the impact of major genes, polygenes, and their interactions on the Vc content of rapeseed seedlings. This provides valuable insights for improving future Vc levels in the future OVDP rapeseed breeding programs. In spite of the advances made in this research, we could not locate the major genes and polygenes on a particular chromosome for now, which needs in depth investigations in future studies.

## 5. Conclusions

Rapeseed (*B. napus* L.) seedlings are a rich source of Vc, with potential benefits for human health. However, the inheritance patterns of this nutritional trait have not yet been fully investigated. An analysis using a major gene plus polygene mixed inheritance model suggests that the Vc content in rapeseed seedlings conforms to the MX2-A-AD model, indicating that it is controlled by two additive major genes plus additive and dominance polygenes. To enhance the Vc content in rapeseed seedlings, a recommended approach involves the use of single cross recross or single backcross methods to transfer major genes, with a more efficient selection expected in the F_2_ generation. In addition, environmental conditions should also be considered in the breeding process. The results of this study provide a foundational basis for the future QTL mapping of nutritional traits and offer valuable insights for targeted breeding to enhance Vc content in rapeseed seedlings. All in all, cultivating high-Vc rapeseed varieties could elevate the nutritional value of “oil–vegetable dual-purpose” rapeseed, which provides consumers with a healthy and nutritious vegetable.

## Figures and Tables

**Table 1:** Statistical analysis of Vc content in six generations of crosses A and B.

Cross	Generation	No. of Plants	Minimum (mg 100 g^−1^)	Maximum (mg 100 g^−1^)	Mean (mg 100 g^−1^)	SD	Variance	CV (%)
Cross A 8S079 × 8S084	P_1_	50	125.02	160.02	141.24 a	8.27	68.37	5.85
	P_2_	50	62.76	90.27	78.58 f	7.26	52.64	9.23
	F_1_	46	96.64	135.68	112.22 c	10.22	104.54	9.11
	F_2_	217	57.70	163.86	101.00 d	20.27	410.95	20.07
	BC_1_P_1_	128	78.25	181.67	125.52 b	23.17	536.94	18.46
	BC_1_P_2_	123	55.30	118.36	87.40 e	14.76	217.78	16.89
Cross B 8S243 × 8S007	P_1_	50	122.79	154.89	139.34 a	9.15	83.73	6.57
	P_2_	50	62.75	89.02	74.30 f	7.30	53.24	9.82
	F_1_	48	99.74	129.13	112.94 c	8.26	68.20	7.31
	F_2_	245	47.23	174.65	94.37 d	23.64	558.84	25.05
	BC_1_P_1_	119	85.32	170.50	122.81 b	19.77	390.70	16.09
	BC_1_P_2_	111	54.54	123.66	87.81 e	16.96	287.71	19.32

Note: values marked with different letters indicate statistically significant differences (*p* < 0.05).

**Table 2:** The skewness and kurtosis values of Vc content in six generations of crosses A and B.

Cross	Generation	P_1_	F_1_	F_1_	F_2_	BC_1_P_1_	BC_1_P_2_
Cross A	Skewness	0.21	−0.18	0.25	0.76	0.36	0.08
	Kurtosis	−0.57	−0.94	−0.96	0.55	−0.60	−0.69
Cross B	Skewness	0.09	0.35	0.12	0.95	0.50	0.18
	Kurtosis	−1.16	−0.54	−1.08	1.35	−0.29	−0.59

**Table 3:** Estimation of the maximum likelihood values and AIC values of 24 different genetic models for crosses A and B.

Model	Maximum Likelihood		AIC Value	
	Cross A	Cross B	Cross A	Cross B
1MG-AD	−2587.82	−2668.37	5183.64	5344.75
1MG-A	−2602.40	−2671.64	5210.79	5349.28
1MG-EAD	−2735.58	−2795.43	5477.15	5596.86
1MG-NCD	−2673.71	−2749.70	5353.42	5505.40
2MG-ADI	−2574.38	−2624.14	5168.76	5268.28
2MG-AD	−2579.41	−2642.25	5170.81	5296.49
2MG-A	−2639.73	−2664.71	5287.45	5337.41
2MG-EA	−2600.38	−2665.74	5206.75	5337.48
2MG-CD	−2711.23	−2768.11	5430.46	5544.22
2MG-EAD	−2711.23	−2768.11	5428.46	5542.22
PG-ADI	−2569.15	−2637.90	5158.29	5295.79
PG-AD	−2596.03	−2681.62	5206.06	5377.24
MX1-AD-ADI	−2555.84	−2627.90	5135.68	5279.79
MX1-AD-AD	−2562.51	−2657.10	5143.03	5332.20
MX1-A-AD	−2572.86	−2649.33	5161.72	5314.66
MX1-EAD-AD	−2595.26	−2677.93	5206.52	5371.87
MX1-NCD-AD	−2573.26	−2657.18	5162.51	5330.35
MX2-ADI-ADI	−2546.93	−2603.42	5129.85	5242.84
MX2-ADI-AD	−2548.58	−2608.82	5127.17	5247.63
MX2-AD-AD	−2561.92	−2636.23	5145.85	5294.46
MX2-A-AD	−2554.48	−2609.53	5126.95	5237.06
MX2-EA-AD	−2572.90	−2635.76	5161.80	5287.52
MX2-CD-AD	−2618.23	−2689.42	5254.46	5396.84
MX2-EAD-AD	−2595.26	−2677.93	5206.51	5371.86

Note: MG: major gene model; MX: mixed major gene and polygene model; PG: polygene model; A: additive effect; D: dominance effect; I: interaction (epistasis); N: negative; E: equal; CD: complete dominance; NCD: negatively complete dominance; EA: equally additive; EAD: equally additive dominance.

**Table 4:** Suitability test of candidate genetic models for Vc content.

Cross	Model	Generation	*U*_1_^2^	*U*_2_^2^	*U*_3_^2^	n*W*^2^	*D*n
Cross A	MX2-A-AD	P_1_	0.10 (0.75)	0.20 (0.66)	0.28 (0.60)	0.10 (0.62)	0.10 (0.60)
		F_1_	0.01 (0.91)	0.13 (0.72)	1.00 (0.32)	0.11 (0.57)	0.11 (0.54)
		P_2_	0.00 (0.96)	0.04 (0.85)	0.89 (0.34)	0.08 (0.71)	0.10 (0.64)
		BC_1_P_1_	0.26 (0.61)	0.06 (0.81)	1.00 (0.32)	0.14 (0.41)	0.08 (0.33)
		BC_1_P_2_	0.34 (0.56)	0.45 (0.50)	0.19 (0.67)	0.06 (0.79)	0.06 (0.77)
		F_2_	0.24 (0.63)	0.37 (0.54)	0.30 (0.58)	0.08 (0.71)	0.61 (0.61)
	MX2-AD-ADI	P_1_	0.15 (0.70)	0.05 (0.83)	0.37 (0.54)	0.11 (0.54)	0.12 (0.42)
		F_1_	0.40 (0.53)	0.12 (0.73)	1.18 (0.28)	0.15 (0.40)	0.15 (0.25)
		P_2_	0.08 (0.78)	0.00 (0.97)	0.88 (0.35)	0.08 (0.68)	0.10 (0.64)
		BC_1_P_1_	0.40 (0.84)	0.01 (0.93)	0.16 (0.69)	0.03 (0.98)	0.05 (0.92)
		BC_1_P_2_	0.24 (0.62)	0.28 (0.60)	0.04 (0.84)	0.05 (0.86)	0.05 (0.91)
		F_2_	0.02 (0.88)	0.01 (0.92)	0.00 (0.85)	0.03 (0.98)	0.04 (0.92)
Cross B	MX2-A-AD	P_1_	0.27 (0.60)	0.60 (0.44)	1.17 (0.28)	0.12 (0.49)	0.10 (0.66)
		F_1_	0.13 (0.71)	0.37 (0.54)	1.05 (0.31)	0.09 (0.65)	0.11 (0.55)
		P_2_	0.27 (0.60)	0.19 (0.66)	0.07 (0.79)	0.07 (0.73)	0.10 (0.63)
		BC_1_P_1_	0.03 (0.86)	0.16 (0.69)	0.89 (0.34)	0.08 (0.68)	0.06 (0.85)
		BC_1_P_2_	1.39 (0.24)	1.27 (0.26)	0.00 (0.96)	0.15 (0.38)	0.07 (0.63)
		F_2_	0.45 (0.50)	0.66 (0.42)	0.43 (0.51)	0.10 (0.58)	0.55 (0.55)
	MX2-ADI-ADI	P_1_	0.01 (0.93)	0.04 (0.85)	1.23 (0.27)	0.11 (0.57)	0.11 (0.48)
		F_1_	0.01 (0.94)	0.03 (0.85)	1.07 (0.30)	0.08 (0.74)	0.10 (0.65)
		P_2_	0.06 (0.81)	0.03 (0.86)	0.05 (0.83)	0.05 (0.88)	0.09 (0.80)
		BC_1_P_1_	0.15 (0.70)	0.04 (0.84)	0.46 (0.50)	0.05 (0.85)	0.05 (0.94)
		BC_1_P_2_	0.00 (0.97)	0.00 (0.99)	0.04 (0.84)	0.02 (1.00)	0.04 (1.00)
		F_2_	0.04 (0.85)	0.09 (0.77)	0.18 (0.67)	0.06 (0.82)	0.05 (0.63)

Note: *U*_1_^2^, *U*_2_^2^, *U*_3_^2^: statistics of the Uniformity test, with the number in parenthesis is a theoretical probability value; n*W*^2^: statistic of Smirnov test; *D*n: statistic of Kolmogorov test. The value in parentheses represents the probability for *U*_1_^2^, *U*_2_^2^, and *U*_3_^2^ and significance levels for n*W*^2^ and *D*n.

**Table 5:** Estimates of the first-order genetic parameters of Vc content.

First-Order Genetic Parameter	Estimate	
	Cross A	Cross B
m	107.11	103.92
*d*_a_	22.43	30.72
*d*_b_	−6.34	−9.61
[*d*]	19.65	15.95
[*h*]	−0.52	3.20
[*h*]/[*d*]	−0.03	0.20

Note: m: mean of graduation; *d*_a_: additive effect of the first major gene; *d*_b_: additive effect of the second major gene; [*d*]: additive effect of polygene; [*h*]: dominant effect of polygene; [*h*]/[*d*]: dominance degree of the polygenes; ‘−’ indicates a negative effect.

**Table 6:** Estimates of the second-order genetic parameters of crosses A and B.

Second-Order Genetic Parameter	Estimate					
	Cross A			Cross B		
	BC_1_P_1_	BC_1_P_2_	F_2_	BC_1_P_1_	BC_1_P_2_	F_2_
*σ^2^_p_*	536.94	217.78	410.95	390.70	281.71	558.84
*σ^2^_e_*	71.93	71.93	71.93	67.07	67.07	67.07
*σ^2^_mg_*	225.76	119.60	339.02	289.72	147.33	491.77
*σ^2^_pg_*	239.25	26.25	0.00	33.91	73.31	0.00
*h^2^_mg_* (%)	42.05	54.92	82.50	74.15	51.21	88.00
*h^2^_pg_* (%)	44.56	12.05	0.00	8.68	25.48	0.00
*h*^2^*_mg + pg_* (%)	86.60	66.97	82.50	82.83	76.69	88.00
1 − *h^2^_mg + pg_* (%)	13.40	33.03	17.50	17.17	23.31	12.00

Note: *σ^2^_p_*: phenotypic variance; *σ^2^_e_*: environmental variance; *σ^2^_mg_*: major gene variance; *σ^2^pg*: polygenic variance; *h^2^_mg_*: major gene heritability; *h^2^_pg_*: polygene heritability.

## Data Availability

Data are contained within the article.

## Supplementary Materials

The following supporting information can be downloaded at: https://www.mdpi.com/article/10.3390/cimb46090568/s1: Figure S1: Chromatogram of vitamin C in rapeseed seedlings; Figure S2: Standard operating curve of vitamin C.
