# Supplementary material for: Genetic Analysis of Vitamin C Content in Rapeseed Seedlings by the Major Gene Plus Polygene Mixed Effect Model
Source: Curr Issues Mol Biol. 2024 Aug 29;46(9):9565–75. doi: 10.3390/cimb46090568 (PMC11429590; doi:10.3390/cimb46090568)
Supplement: Supplementary file 1 [file cimb-46-00568-s001.zip › cimb-3169549-supplementary.pdf]

Supplementary Materials

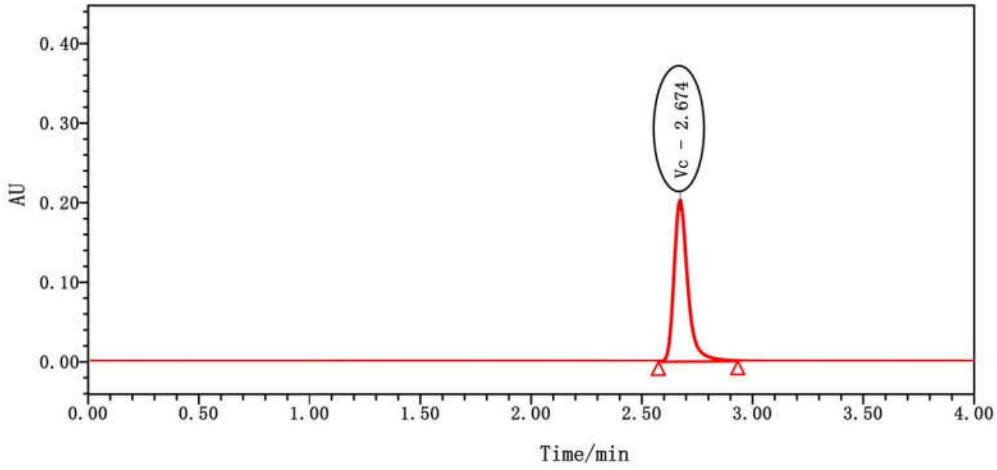

Figure S1. Chromatogram of vitamin C in rapeseed seedlings. Note: The x-axis represents peak time, and the y-axis represents absorbance units (AU).

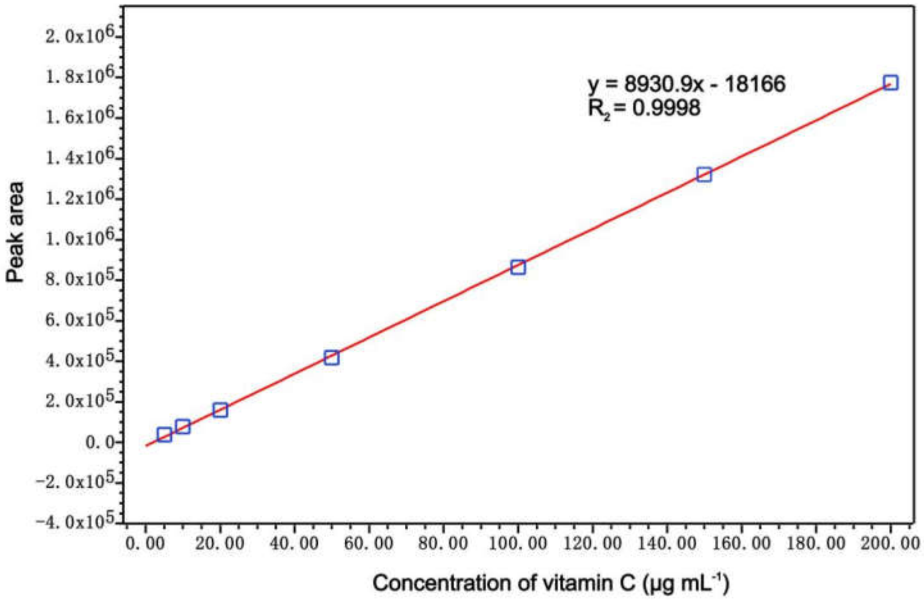

Figure S2. Standard operating curve of vitamin C. Note: In the regression equation, x represents concentration of the standard vitamin C solution, and y represents the peak area.
